# Targeting Angiogenesis in Breast Cancer: Current Evidence and Future Perspectives of Novel Anti-Angiogenic Approaches

**DOI:** 10.3389/fphar.2022.838133

**Published:** 2022-02-25

**Authors:** Nehad M. Ayoub, Sara K. Jaradat, Kamal M. Al-Shami, Amer E. Alkhalifa

**Affiliations:** ^1^ Department of Clinical Pharmacy, Faculty of Pharmacy, Jordan University of Science and Technology (JUST), Irbid, Jordan; ^2^ Department of Drug Discovery and Development, Harrison School of Pharmacy, Auburn University, Auburn, AL, United States

**Keywords:** breast cancer, angiogenesis, tumor vasculature, VEGF, tyrosine kinase inhibitor (TKI), resistance

## Abstract

Angiogenesis is a vital process for the growth and dissemination of solid cancers. Numerous molecular pathways are known to drive angiogenic switch in cancer cells promoting the growth of new blood vessels and increased incidence of distant metastasis. Several angiogenesis inhibitors are clinically available for the treatment of different types of advanced solid cancers. These inhibitors mostly belong to monoclonal antibodies or small-molecule tyrosine kinase inhibitors targeting the classical vascular endothelial growth factor (VEGF) and its receptors. Nevertheless, breast cancer is one example of solid tumors that had constantly failed to respond to angiogenesis inhibitors in terms of improved survival outcomes of patients. Accordingly, it is of paramount importance to assess the molecular mechanisms driving angiogenic signaling in breast cancer to explore suitable drug targets that can be further investigated in preclinical and clinical settings. This review summarizes the current evidence for the effect of clinically available anti-angiogenic drugs in breast cancer treatment. Further, major mechanisms associated with intrinsic or acquired resistance to anti-VEGF therapy are discussed. The review also describes evidence from preclinical and clinical studies on targeting novel non-VEGF angiogenic pathways in breast cancer and several approaches to the normalization of tumor vasculature by targeting pericytes, utilization of microRNAs and extracellular tumor-associate vesicles, using immunotherapeutic drugs, and nanotechnology.

## Introduction

Angiogenesis is the process of the formation of new blood vessels from pre-existing ones (Teleanu et al., 2019). It plays a key role in several physiologic and pathologic processes (Ramjiawan et al., 2017; Nowak-Sliwinska et al., 2018). Angiogenesis is tightly regulated by the interplay between pro- and anti-angiogenic factors (Rust et al., 2019). Several pro-angiogenic factors are known to drive vascular growth including vascular endothelial growth factor (VEGF), fibroblast growth factor (FGF), platelet-derived growth factor (PDGF), angiopoietins (Angs), hepatocyte growth factor (HGF), transforming growth factor-β (TGF-β), and matrix metalloproteinases (MMPs). Among these factors, the VEGF family is considered a major regulator of vascular growth and angiogenesis (Rust et al., 2019). The VEGF family includes VEGF-A (also known as VEGF), VEGF-B, VEGF-C, VEGF-D, and placental growth factor (Yang et al., 2018). These ligands bind to their endothelial VEGF receptors (VEGFRs); VEGFR-1, VEGFR-2, and VEGFR-3, which belong to the family of receptor tyrosine kinases (RTKs) (Yang et al., 2018). Anti-angiogenic factors include thrombospondin-1, angiostatin, endostatin, vasostatin, tumstatin, interferon-γ, glycosaminoglycan, anti-tissue factor/anti-factor VIIa, and tissue inhibitors of MMPs (Rust et al., 2019). Disturbances in the balance between pro- and anti-angiogenic factors can drive pathologic angiogenesis (Lugano et al., 2020).

### Physiologic Angiogenesis: Process and Types

Blood vessels of the microvasculature are composed of two major cell types: endothelial cells and perivascular cells known as pericytes (Karamysheva, 2008). Pericytes are known for regulating endothelial cell proliferation, differentiation, and migration through paracrine regulators and vasoactive agents (Kelly-Goss et al., 2014). Under physiologic conditions, endothelial cells exist in a quiescent non-proliferative state (Kruger-Genge et al., 2019). However, in response to vascular injury, inflammation, or hypoxia, angiogenesis is induced through a cascade of highly regulated sequential events (Kruger-Genge et al., 2019). Quiescent endothelial cells are initially activated through increased levels of pro-angiogenic factors (Kruger-Genge et al., 2019). During the activation phase, pericytes are detached from the vessel wall and blood vessels dilate and tight junctions of endothelial cells are disrupted allowing endothelial cells to proliferate and elongate to form the new blood vessel (Mazurek et al., 2017). Simultaneously, proteases remodel the interstitial matrix enabling endothelial cell migration and fusion of newly developed blood vessels (Carmeliet and Jain, 2011). Subsequently, the proliferative activity of endothelial cells is reduced to restore the quiescent state of endothelial cells, and pericytes are recruited to the newly formed blood vessel (Rust et al., 2019). The interaction between endothelial cells and pericytes during angiogenesis is regulated, in part, by Ang-1/Tie-2, TGF-β, and PDGF signaling (Kelly-Goss et al., 2014). The presence of pericyte coverage of endothelial cells supports the maturation and stabilization of blood vessels (Gerhardt and Betsholtz, 2003). Table 1 summarizes the activity of major pro-angiogenic factors.

**TABLE 1:** Angiogenic activity of major families of pro-angiogenic factors.

Angiogenic factors	Target receptor(s)	Angiogenic activity
Vascular endothelial growth factor (VEGF) family		
VEGF-A	VEGFR-1	Promote the formation of primitive tubular structures at early stage of angiogenesis. Modulate endothelial cell proliferation, migration, metabolic homeostasis, and tubulogenesis
VEGF-B		
VEGF-C	VEGFR-2	
VEGF-D	VEGFR-3	
PlGF		
Fibroblast growth factor (FGF) family		
FGF-1 (acidic)	FGFR-1	Induce secretion of MMPs, activation of plasminogen, and collagenase responsible for the degradation and organization of extracellular matrix. Induce proliferation and physical organization of endothelial cells into tube-like structures
	FGFR-2	
	FGFR-3	
FGF-2 (basic)	Integrins	
Platelet-derived growth factor (PDGF) family		
PDGF-A	PDGFR-α	Promote vessel maturation and recruit smooth muscle cells and pericytes to newly formed vessels
PDGF-B		
PDGF-C	PDGFR-β	
PDGF-D		
Hepatocyte growth factor (HGF) family		
HGF	MET	Promote proliferation, migration, invasion, branching morphogenesis, and capillary tube organization
Transforming growth factor (TGF-β) family		
TGF-β1	TGFβR-1	Stimulate the production of extracellular matrix and regulate the interaction between endothelial cells, and mural cells
TGF-β2		
TGF-β3	TGFβR-2	
Angiopoietins (Ang) family		
Ang-1	Tie-1	Ang-1 promotes vessel maturation and stabilization of the newly formed vessels and Ang-2 induces vessel destabilization, pericytes detachment, vessel sprouting, and angiogenesis
Ang-2	Tie-2	

FGFR, FGF receptor; MMP, matrix metalloproteinase; PDGFR, PDGF receptor; PlGF, placental growth factor; TGFβR, TGFβ receptor; VEGF, vascular endothelial growth factor; VEGFR, VEGF receptor.

Two basic types of angiogenesis exist, sprouting and intussusception (Adair TH, 2010). Sprouting angiogenesis represents the major mechanism of angiogenic growth and is characterized by sprouts of endothelial cells growing through the branching morphogenesis process (Teleanu et al., 2019; Lugano et al., 2020). Alternatively, intussusception angiogenesis involves the *splitting* of existing blood vessels to form new ones (Adair TH, 2010).

### Tumor Vasculature

The role of angiogenesis in cancer growth and metastasis was first introduced by Judah Folkman who described the growth of blood vessels as an essential process for the growth of solid tumors (Folkman, 1971). Angiogenesis establishes vascular networks to supply oxygen and nutrients essential for tumor growth and metastasis (Lugano et al., 2020). Tumors secrete various pro-angiogenic factors to promote the sprouting of new blood vessels from existing vasculature thus enabling tumor growth and metastatic spreading to distant organs (Yonenaga et al., 2005). Though tumor blood vessels carry distinct molecular markers in the endothelium, several other markers are shared by vessels in non-malignant tissues (Ruoslahti, 2002).

Although angiogenesis plays a key role in tumor vascular growth, non-angiogenic mechanisms of vascularization exist to meet the demands for oxygen and nutrients by tumors (Stessels et al., 2004). Vasculogenesis is the process of the formation of blood vessels from circulating cells (Brown, 2014). The main driver of vasculogenesis is the stromal cell-derived factor-1 (SDF1/CXCL12) upregulated in response to tumor hypoxia and increased levels of hypoxia-inducible factor-1 (HIF-1) (Brown, 2014). Vasculogenesis is mediated by the recruitment of endothelial progenitor cells (EPCs) or bone marrow-derived hematopoietic cells leading to the formation of new blood vessels in the tumor microenvironment (Lugano et al., 2020). EPCs may originate from hematopoietic stem cells, myeloid cells, circulating mature endothelial cells, or other circulating progenitor cells. Regularly, VEGF in the tumor microenvironment mobilizes VEGFR-2-positive EPCs from the bone marrow to initiate vasculogenesis (Lugano et al., 2020). In addition, cancer cells themselves have unique characteristics to form vessel-like channels within the tumor in a process known as vascular mimicry (Ruoslahti, 2002). These vascular structures lack endothelial cells and serve as alternate channels to supply blood and nutrients to tumor cells (Lugano et al., 2020). Like in the case of vasculogenesis, hypoxia promotes vascular mimicry (Andonegui-Elguera et al., 2020). Vascular co-option has also been found to be an important approach to establish tumor vasculature, especially in the more aggressive types of tumors (Qian et al., 2016). In the latter procedure, tumor cells obtain blood supply by hijacking blood vessels in the surrounding normal tissue along with the migration and invasion of cancer cells (Donnem et al., 2013; Qian et al., 2016). Further, cancer stem cells trans-differentiation to endothelial cells and vascular smooth muscle-like cells has been observed in different types of tumors to promote tumor vascularization (Lugano et al., 2020).

**FIGURE 1:**
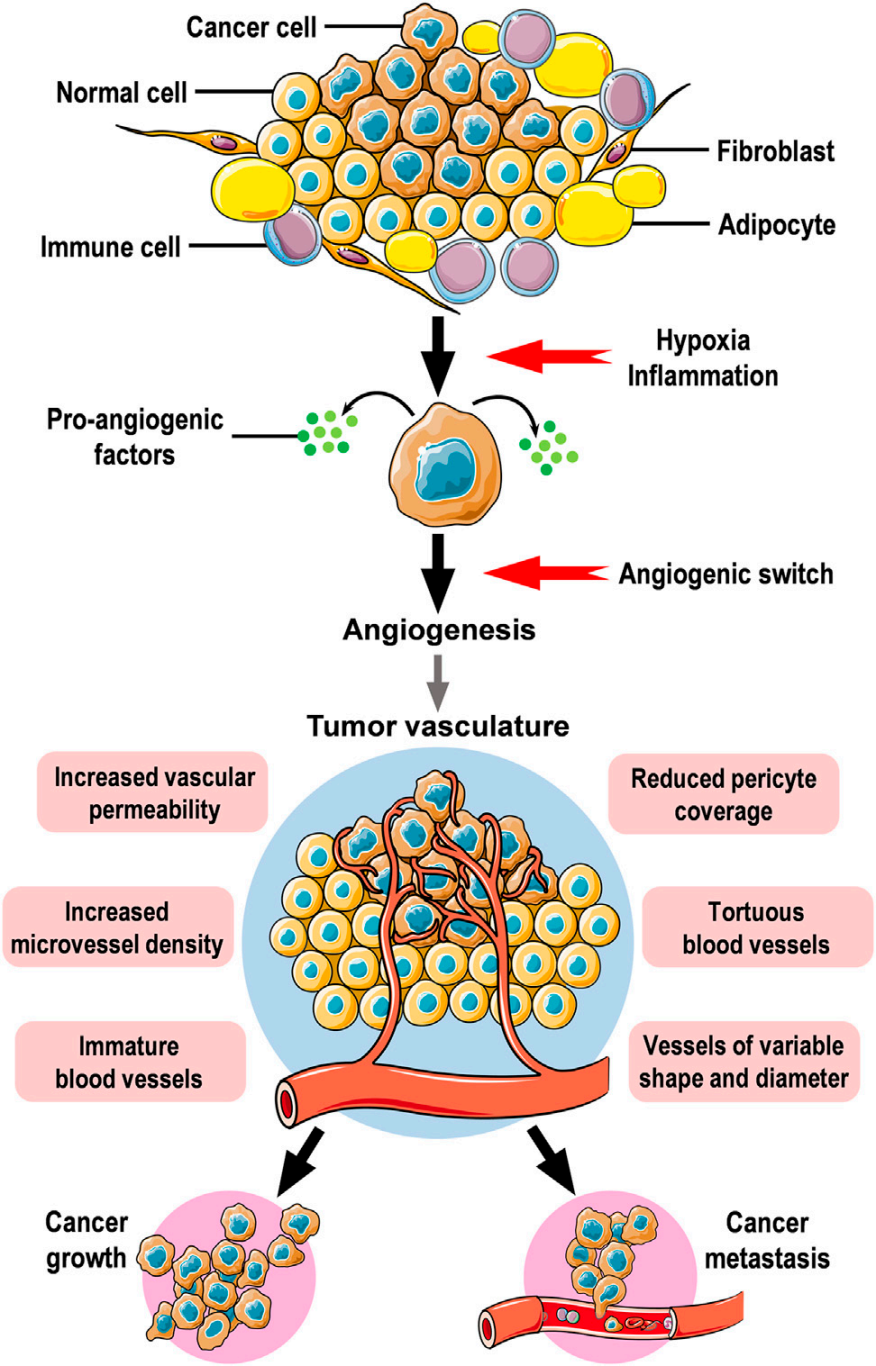
Angiogenesis and tumor vasculature. Cancer cells are regularly surrounded by stromal cells in the tumor microenvironment. Stromal cells include fibroblasts, immune cells, and adipocytes which are common components in the stroma surrounding mammary epithelium. Hypoxic conditions, inflammatory cytokines, and growth factors secreted by stromal cells drive neoplastic cells to secrete pro-angiogenic factors which will affect nearby blood vessels to induce angiogenic activity leading to the formation of new blood vessels to supply oxygen and nutrients to tumor cells. The newly formed tumor vasculature is dynamically unstable, hyperpermeable, immature with reduced pericyte coverage, and irregular. Angiogenesis is an essential step for the growth and distant metastasis of solid cancers. The figure was created using free medical images available from Servier Medical Art at: smart.servier.com.

Unlike normal blood vessels, tumor vasculature displays multiple functional and structural abnormalities characterized by unusual leakiness, high tortuosity, and poor coverage by pericytes (Dudley, 2012; Goel et al., 2013). These abnormalities mediate chaotic blood flow and support the hematogenous dissemination of tumor cells while impairing the delivery of chemotherapeutic drugs (Goel et al., 2013). Though the measurement of microvascular density is the gold standard approach for quantification of angiogenesis (Tahergorabi and Khazaei, 2012), the maturity and stability of blood vessels are being increasingly recognized in the assessment of tumor vasculature (Fakhrejahani and Toi, 2012). Intratumoral hypoxia triggers the formation of dysfunctional blood vessels thus facilitating cancer cell metastasis and reducing the efficacy of treatments (Kugeratski et al., 2019). Hypoxia increases cell adhesion, coagulant properties, endothelial intracellular gaps, and endothelial permeability, all of which are crucial for the processes of intravasation, and extravasation needed for cancer cell metastasis (Evans et al., 2012). Endothelial cells exposed to hypoxia demonstrated an amplified pro-inflammatory phenotype, characterized by an increased expression of inflammatory cytokines (Tellier et al., 2015). In the microenvironment of solid tumors, hypoxia has been shown to stimulate autophagy in tumor-associated blood vessels which in turn can alter metabolic pathways and surface markers of endothelial cells (Verhoeven et al., 2021). Additionally, hypoxic cancer-associated fibroblasts induced blood vessel abnormalities by altering the secretion of various pro- and anti-angiogenic factors leading to changes in endothelial cell function and promoting angiogenesis (Kugeratski et al., 2019). Figure 1 illustrates angiogenic activity in the tumor microenvironment.

Targeting angiogenesis in cancer therapy is an appealing approach to stop the growth and metastasis of solid cancers. Nevertheless, clinical evidence showed variable sensitivity to angiogenesis inhibitors over different tumor types. The main objectives to conceive and prepare this review paper were to 1) provide a summary of the state of angiogenesis inhibitors in the treatment of breast cancer, 2) analyze the factors attributing to the lack of efficacy of anti-angiogenic drugs, 3) explore new potential drug targets for angiogenesis inhibitors through non-VEGF/VEGFR signaling, and 4) describe novel approaches for targeting tumor vascularization and their potential implementation in breast cancer. The next part of this review describes available evidence for the effect of angiogenesis inhibitors in breast cancer treatment.

## Breast Cancer and Angiogenesis: The Status of Angiogenesis Inhibitors

Breast cancer is the most common malignancy among women worldwide (Siegel et al., 2020). It is a heterogeneous disease that is further classified into different molecular subtypes based on gene expression profiling (Polyak, 2007). The molecular subtypes include luminal A, luminal B, human epidermal growth factor receptor 2 (HER2)-positive, and basal-like breast cancer (Polyak, 2007). These subtypes have distinct pathologic features and clinical outcomes (Polyak, 2007). Luminal tumors express hormone receptors and luminal epithelial elements of the breast (Dai et al., 2015). The HER2-positive tumors are characterized by overexpression of HER2, while the basal-like tumors compose a large group of triple-negative breast cancer (TNBC) lacking expression of hormone receptors and HER2 (Dai et al., 2015).

Metastatic breast cancer is a major cause of morbidity and mortality among patients diagnosed with the disease (Chen W. et al., 2018). About 20–30% of patients with early-stage breast cancer will develop metastatic disease (Chen W. et al., 2018). Breast cancer cells commonly metastasize to bone, liver, lung, and brain (Chen W. et al., 2018). Patterns of metastatic disease are related to the molecular subtype and could result in distinct survival outcomes (Kozlowski et al., 2015; Chen W. et al., 2018). Despite advancements in breast cancer therapeutics, prevention of disease recurrence and metastasis is a challenge to oncologists. Cancer metastasis involves a cascade of sequential, multistep, and multifunctional biological events which eventually lead to the spread of cancer cells from the primary tumor site to distant sites (Kozlowski et al., 2015). In this complex process, angiogenesis is an essential early step in the metastatic cascade (Kozlowski et al., 2015).

Experimental and clinical studies revealed that VEGF is the predominant angiogenic factor in breast cancer (Niu and Chen, 2010; Ribatti et al., 2016). Overexpression of VEGF occurs frequently before the invasion of breast cancer cells (Schneider and Sledge, 2007). VEGF expression correlated with inferior outcomes in breast cancer (Schneider and Miller, 2005). Serum VEGF levels correlated with an advanced stage of breast cancer (Ribatti et al., 2016; Raghunathachar Sahana et al., 2017). Studies have also found an inverse relationship between VEGF expression and overall survival (OS) in both node-positive and node-negative disease (Ribatti et al., 2016). Angiogenesis in breast carcinoma has been also found to be regulated by VEGFR-2, VEGFR-3, VEGF-D, and VEGF-C (Longatto Filho et al., 2010; Eroglu et al., 2017). Expression of VEGF-D was associated with lymph node metastasis in breast cancer tissues (Eroglu et al., 2017). Besides VEGF, multiple pro-angiogenic factors are expressed by invasive human breast cancer including TGF-β1, pleiotrophin, acidic and basic FGF, placental growth factor, and PDGF (Relf et al., 1997). High microvessel density was further associated with invasive carcinoma and correlated with a greater likelihood of metastatic disease and shorter OS in breast cancer patients (Schneider and Miller, 2005). In addition, non-angiogenic pathways of vascularization were reported in breast cancer. Shirakawa et al. indicated the existence of vasculogenesis in breast cancer (Shirakawa et al., 2001; Shirakawa et al., 2002). Vascular mimicry and co-option were also observed and were associated with poor prognosis and increased metastasis of breast tumors, respectively (Stessels et al., 2004; Andonegui-Elguera et al., 2020). Collectively, angiogenic and non-angiogenic vascularization pathways may co-exist in the breast cancer microenvironment.

Inhibitors of angiogenesis classically prevent the expression or block the activity of pro-angiogenic factors secreted by tumor cells by targeting their receptors on endothelial cells (El-Kenawi and El-Remessy, 2013). Accordingly, angiogenesis inhibitors deprive tumors of nutrients necessary for growth and promote normalization of tumor vasculature to improve the delivery of cytotoxic chemotherapy (El-Kenawi and El-Remessy, 2013). Despite the favorable outcomes of using angiogenesis inhibitors in different types of solid tumors, these drugs have failed to provide a survival advantage in breast cancer. The next part summarizes results from clinical trials investigating angiogenesis inhibitors in patients with breast cancer.

### Bevacizumab

Bevacizumab (Avastin^®^) is a humanized anti-VEGF monoclonal antibody approved in combination with chemotherapy for the treatment of several advanced solid cancers (Kazazi-Hyseni et al., 2010). It binds selectively to circulating VEGF, thereby inhibiting VEGF binding to its receptor (Kazazi-Hyseni et al., 2010). The AVF2119G clinical trial was the first to provide published data regarding the clinical usefulness of bevacizumab in the treatment of metastatic breast cancer (Miller et al., 2005). The results of this phase III randomized trial showed that the addition of bevacizumab to capecitabine in second-line therapy improved response rate compared to capecitabine treatment alone (19.8 vs. 9.1%, *p* = 0.001) (Miller et al., 2005). However, this combination neither improved progression-free survival (PFS) (median, 4.86 vs. 4.17 months) nor OS (median, 15.1 vs. 14.5 months) (Miller et al., 2005). The E2100 was an open-label, randomized, phase III clinical trial that investigated the efficacy and safety of the combination of paclitaxel and bevacizumab compared to paclitaxel as a first-line treatment for metastatic breast cancer (Miller et al., 2007). Findings from the E2100 trial indicated a significantly improved PFS for the combination arm compared to paclitaxel alone, while OS was similar in both treatment arms (median, 25.2 vs. 26.7 months; *p* = 0.16) (Miller et al., 2007). The results from the AVF2119G and E2100 trials granted accelerated approval of bevacizumab use in the treatment of metastatic breast cancer by the US Food and Drug Administration (FDA). Subsequently, several phase III clinical trials have evaluated bevacizumab with chemotherapy revealing no significant improvement in OS (Miles et al., 2010; Brufsky et al., 2011; Robert et al., 2011). The failure of achieving a survival advantage along with serious tolerability issues created controversy over the real value of bevacizumab treatment in metastatic breast cancer and further brought its approval into question. Ultimately, the US FDA had revoked the indication of bevacizumab to treat patients with metastatic breast cancer in 2011 (Sasich and Sukkari, 2012).

After the withdrawal statement, results from other clinical trials on the use of bevacizumab in breast cancer were published. The BEATRICE study was an open-label, randomized, phase III clinical trial that assessed the addition of bevacizumab to chemotherapy in adjuvant settings in patients with operable TNBC (Cameron et al., 2013). Results from the BEATRICE study revealed no improvement of OS compared to patients receiving chemotherapy alone (Cameron et al., 2013; Bell et al., 2017). Rather, grade III adverse events were increased in the bevacizumab arm (Cameron et al., 2013). The E5103 study was a double-blind, phase III trial of adjuvant chemotherapy with and without bevacizumab in breast cancer patients with lymph node-positive and high-risk lymph node-negative disease (Miller et al., 2018). The findings of the study failed to show improvements in invasive disease-free survival or OS upon the addition of bevacizumab to chemotherapy in adjuvant settings in breast cancer patients with high-risk HER2-negative disease (Miller et al., 2018).

Other studies investigated the value of adding bevacizumab to chemotherapy in the neoadjuvant treatment of breast cancer (Bear et al., 2012; von Minckwitz et al., 2012; Clavarezza et al., 2013). The primary endpoint in these studies was the pathologic complete response (pCR) rate. Overall, findings from these clinical trials showed a favorable response for using bevacizumab with chemotherapy in neoadjuvant treatment in terms of increased pCR rates (Bear et al., 2012; von Minckwitz et al., 2012; Clavarezza et al., 2013; Tampaki et al., 2018).

Recently, Martin et al. examined the addition of bevacizumab to endocrine drugs as first-line treatment in metastatic hormone receptor-positive breast cancer through pooled data analysis from the LEA and CALGB 40503 trials (Martin et al., 2019). The addition of bevacizumab significantly improved PFS compared to endocrine treatment alone, however, there was no difference in OS between both groups. Besides, a significantly higher rate of grade III/IV adverse events was observed for the combination treatment (Martin et al., 2019). Besides, a phase II trial of nab-paclitaxel and bevacizumab, followed by maintenance therapy with bevacizumab and erlotinib, for patients with metastatic TNBC was conducted by Symonds et. al. (Symonds et al., 2019). No significant difference was seen for either PFS or OS for patients enrolled and none of them achieved complete response (Symonds et al., 2019).

### Ramucirumab

Ramucirumab (Cyramza^®^) is a monoclonal antibody targeting VEGFR-2 (Singh and Parmar, 2015). It was first approved by the US FDA in 2014 as monotherapy for the treatment of metastatic gastric cancer (Casak et al., 2015). Ramucirumab approval was thereafter expanded to combination treatment with chemotherapy for gastric cancer, non-small cell lung cancer (NSCLC), and colon cancer. The drug is also approved as monotherapy for hepatocellular carcinoma (HCC), and most recently in combination with erlotinib for patients with epidermal growth factor receptor (EGFR)-positive NSCLC (Effing and Gyawali, 2020).

Few clinical trials investigated ramucirumab treatment in breast cancer. In phase II, randomized, open-label study, the addition of ramucirumab to capecitabine in previously treated patients with locally advanced and metastatic breast cancer failed to improve PFS and OS compared to capecitabine therapy alone (Vahdat et al., 2017). The frequency of adverse effects was increased in the combination group and included headache, anorexia, constipation, epistaxis, and hypertension (Vahdat et al., 2017). Another phase II, randomized, open-label clinical trial revealed no difference in survival for the combination of ramucirumab and eribulin versus eribulin monotherapy in patients with advanced breast cancer (Yardley et al., 2016b). The ROSE/TRIO-12 was a randomized, placebo-controlled, phase III trial evaluating the addition of ramucirumab to first-line docetaxel treatment in HER2-negative metastatic breast cancer (Mackey et al., 2015). In agreement with findings from previous phase II trials, ramucirumab neither improved PFS nor OS compared to docetaxel treatment (median, 9.5 vs. 8.2 months; *p* = 0.077 and 27.3 vs. 27.2 months; *p* = 0.915, respectively). Higher rates of toxicity were reported in patients receiving ramucirumab treatment (Mackey et al., 2015).

### Tyrosine Kinase Inhibitors

Tyrosine kinase inhibitors (TKIs) are small molecules that inhibit the kinase domain of RTKs thus inhibiting receptor activation and downstream signaling (Fakhrejahani and Toi, 2014). Several angiogenesis inhibitors are small-molecule TKIs.

#### Sorafenib

Sorafenib (Nexavar^®^) is an oral multikinase inhibitor of VEGFR, PDGF receptor (PDGFR), and Raf (Ben Mousa, 2008). The drug is approved for the treatment of renal cell carcinoma (RCC) (Kane et al., 2006), HCC (Kane et al., 2009), and thyroid cancer (Pitoia and Jerkovich, 2016). The use of sorafenib in breast cancer showed modest efficacy in early clinical trials. In a randomized, double-blind, placebo-controlled, phase IIb trial, the addition of sorafenib to capecitabine showed higher toxicity and no improvement in OS in patients with locally advanced or metastatic HER2-negative breast cancer (Baselga et al., 2012). In another phase IIb, double-blind, randomized, placebo-controlled study, the addition of sorafenib to capecitabin or gemcitabine moderately improved PFS compared to placebo and chemotherapy in breast cancer patients (median, 3.4 vs. 2.7 months; *p* = 0.02) (Schwartzberg et al., 2013). Nevertheless, no significant effect was observed for OS for patients in the sorafenib arm (Schwartzberg et al., 2013). Despite the modest effects for sorafenib treatment observed in terms of improved PFS, subsequent clinical trials failed to confirm the survival advantage for the drug in breast cancer treatment. The RESILIENCE study, a randomized, double-blind, placebo-controlled, phase III trial, revealed a lack of survival advantage for combining sorafenib with capecitabine or placebo in patients with locally advanced or metastatic HER2-negative breast tumors (Baselga et al., 2017). Nevertheless, rates of grade III toxicities were notably higher in the sorafenib arm (Baselga et al., 2017). In the PASO trial, the safety and efficacy of adding sorafenib to paclitaxel compared to paclitaxel alone were assessed in an open-label, randomized, phase II study design in patients with locally advanced or metastatic HER2-negative breast cancer (Decker et al., 2017). Interestingly, a pre-planned efficacy interim analysis showed that patients on paclitaxel monotherapy had a significantly greater PFS and OS compared to patients in the combination arm. Toxicities were higher in the combination arm and the study was therefore discontinued (Decker et al., 2017). Similarly, results from the MADONNA study, a multicenter, double-blind, phase II study, revealed a lack of survival advantage upon the addition of sorafenib to docetaxel as first-line treatment in breast cancer patients with metastatic or locally advanced HER2-negative disease (Mavratzas et al., 2019). Recently, Ianza et al. showed no difference in survival outcomes for adding sorafenib to letrozole and cyclophosphamide in postmenopausal patients with locally advanced estrogen receptor (ER)-positive, HER2-negative breast cancer in a phase III trial (Ianza et al., 2020). Interestingly, a higher percentage of patients on sorafenib treatment had disease progression (Ianza et al., 2020). Other clinical studies have constantly supported a lack of survival advantage for the combination of sorafenib and chemotherapy in patients with advanced breast cancer (Gradishar et al., 2013; Luu et al., 2014; Yardley et al., 2016a).

#### Sunitinib

Sunitinib (Sutent^®^) is a novel oral multitargeted TKI of VEGFR-1, VEGFR-2, fetal liver tyrosine kinase receptor 3, c-KIT, PDGFR-α, and PDGFR-β (Le Tourneau et al., 2007). Sunitinib is FDA approved for the treatment of advanced RCC, gastrointestinal stromal tumors (Le Tourneau et al., 2007), and advanced pancreatic neuroendocrine tumors (Blumenthal et al., 2012). Clinical studies for the efficacy of sunitinib in breast cancer treatment have produced disappointing results (Yardley et al., 2012). In 2010, a multicenter, randomized, open-label, phase III trial was conducted to assess the effect of sunitinib versus capecitabine as a treatment for advanced breast cancer patients with disease recurrence after anthracycline and taxane therapy (Barrios et al., 2010). Compared to the capecitabine arm, PFS and OS were shorter for sunitinib (median, 2.8 vs. 4.2 months; and 15.3 vs. 24.6 months, respectively) (Barrios et al., 2010). Sunitinib treatment was also associated with a higher rate and severity of adverse events compared with capecitabine.

Bergh et al. demonstrated no improvement in PFS or OS in breast cancer patients treated with the combination of sunitinib and docetaxel compared to docetaxel alone in an open-label, randomized, phase III trial (Bergh et al., 2012). Moreover, more adverse events, deaths, and treatment discontinuations were observed in the combination arm (Bergh et al., 2012). In a randomized, phase II trial comparing sunitinib to the standard of care in patients with TNBC who relapsed after anthracycline- and taxane-based chemotherapy, no difference between both treatment arms for PFS and OS was observed (Curigliano et al., 2013). In a randomized, phase III study by Crown et al., sunitinib and capecitabine treatment was compared to capecitabine alone in metastatic breast cancer patients who received prior chemotherapy (Crown et al., 2013). The combination arm failed to improve therapeutic outcomes compared to the capecitabine arm as no statistically significant difference was observed for PFS (median, 5.5 vs. 5.9 months; *p* = 0.941) and OS (median, 16.4 vs. 16.5 months; *p* = 0.484) (Crown et al., 2013). In addition, the combination of sunitinib with neoadjuvant chemotherapy in patients with locally advanced or metastatic breast cancer did not improve pCR and was not recommended based on results from phase I/II clinical trials (Yardley et al., 2015; Wong et al., 2016).

#### Vandetanib

Vandetanib (Caprelsa^®^) is an oral small-molecule inhibitor of VEGFR-2, VEGFR-3, EGFR, and RET (Chau and Haddad, 2013). It is approved for the treatment of medullary thyroid carcinoma (Chau and Haddad, 2013). The efficacy and safety of vandetanib with docetaxel as a second-line treatment for advanced breast cancer was assessed in a double-blind, placebo-controlled, randomized, phase II study (Boer et al., 2012). Though well-tolerated, the combination of vandetanib and docetaxel did not improve outcomes compared to placebo and docetaxel (Boer et al., 2012). Clemons et al. also showed no difference in PFS or OS for the combination of vandetanib and fulvestrant compared to placebo in postmenopausal patients with metastatic breast cancer in a phase II trial (Clemons et al., 2014).

#### Axitinib

Axitinib (Inlyta^®^) is an oral second-generation pan-VEGFR TKI (Bellesoeur et al., 2017). The drug is approved for the treatment of advanced RCC (Tyler, 2012). Rugo et al. assessed the efficacy of axitinib plus docetaxel compared to docetaxel and placebo in metastatic breast cancer in a randomized, double-blind, phase II study (Rugo et al., 2011). The addition of axitinib to capecitabine did not significantly improve time to progression compared to the placebo arm (median, 8.1 vs. 7.1 months; *p* = 0.156). Toxicity was increased in the axitinib and docetaxel arm, and most grade III/IV adverse events included diarrhea, fatigue, stomatitis, mucositis, asthenia, and hypertension (Rugo et al., 2011).

#### Pazopanib

Pazopanib (Votrient^®^) is an oral multitarget TKI of VEGFR, PDGFRs, FGFR, and c-KIT (Lee et al., 2019). It is approved for the treatment of advanced RCC and advanced soft-tissue sarcoma (Nguyen and Shayahi, 2013). In 2010, results from a phase II study of single-agent pazopanib in patients with recurrent or metastatic breast cancer revealed promising activity in terms of disease stability and tolerable adverse events (Taylor et al., 2010). Nevertheless, subsequent phase II trials for the combination of lapatinib and pazopanib in HER2-positive breast cancer failed to show survival advantage compared to lapatinib alone. The combination also had increased toxicity compared to lapatinib monotherapy (Cristofanilli et al., 2013; Johnston et al., 2013). The addition of pazopanib to chemotherapy in neoadjuvant treatment for HER2-negative locally advanced breast cancer was assessed in a phase II study, however substantial toxicity resulted in a high discontinuation rate of pazopanib (Tan et al., 2015).

#### Cediranib

Cediranib (Recentin^®^) is a pan-VEGFR inhibitor (Tang et al., 2017). It has been assessed in combination with hormonal treatments in breast cancer patients. A randomized, phase II study evaluated cediranib plus fulvestrant in postmenopausal women with hormone-sensitive metastatic breast cancer compared to placebo (Hyams et al., 2013). The addition of cediranib to fulvestrant did not improve median PFS versus placebo. Furthermore, the rates of grade III adverse events, discontinuations, and dose reductions were higher in the cediranib arm (Hyams et al., 2013).

## Proposed Mechanisms for the Failure of Angiogenesis Inhibitors in Breast Cancer

Evidence from clinical trials constantly showed disappointing treatment outcomes for targeting VEGF/VEGFR signaling in breast cancer. The available experimental evidence, which is not yet definitive, proposes several distinct mechanisms that manifest tumor rescue pathways to anti-angiogenic therapies. Several mechanisms for intrinsic and acquired resistance to angiogenesis inhibitors have now been explored. Some of these mechanisms are discussed below.

### Upregulation of Alternative Angiogenic Pathways

The activation of compensatory pro-angiogenic pathways in response to anti-VEGF therapy is a well-established mechanism of acquired resistance in tumors (Bergers and Hanahan, 2008; Ramadan et al., 2020). Blockade of the VEGF/VEGFR signaling pathway can aggravate hypoxia resulting in the upregulation of alternative angiogenic factors such as PDGFs, FGFs, chemokines, interleukin-8 (IL-8), Delta-like ligand-4, and ephrins (Bergers and Hanahan, 2008; Lord and Harris, 2010; Carmeliet and Jain, 2011). Collectively, these angiogenic factors may rescue tumor vascularization despite the presence of the VEGF/VEGFR inhibitor (Bergers and Hanahan, 2008; Carmeliet and Jain, 2011; Ramadan et al., 2020).

### Recruitment of Vascular Progenitors

Typically, vasculogenesis is a minor pathway in the development of tumor vasculature at which angiogenesis is the primary pathway. However, upon the inhibition of angiogenic growth, vasculogenesis may become crucial to maintaining tumor vasculature (Brown, 2014). Hypoxia-induced by anti-VEGF therapy leads to the recruitment of pro-angiogenic bone marrow-derived cells (BMDCs) to the tumor microenvironment (Lord and Harris, 2010; Ramadan et al., 2020). BMDCs can restore vascularization of tumors thus enabling them to overcome hypoxia and become resistant to anti-VEGF drugs (Bergers and Hanahan, 2008; Lord and Harris, 2010). Several BMDCs have been identified in the tumor microenvironment such as tumor-associated macrophages (TAMs), pro-angiogenic monocytic cells, myeloid cells, and Tie-2-expressing macrophages (Lord and Harris, 2010). TAMs were associated with high VEGF expression and high microvessel density in ductal breast carcinoma (Longatto Filho et al., 2010). Tripathi et al. revealed that TAMs were recruited to tumor microenvironment in an animal model of breast cancer by eotaxin and oncostatin M cytokines (Tripathi et al., 2014). Blocking these cytokines with neutralizing antibodies reduced tumor vascularization and improved sensitivity to bevacizumab (Tripathi et al., 2014). Liu et al. demonstrated that inhibiting SDF1/CXCL12 with a neutralizing antibody decreased infiltration of myeloid cells and correlated with reduced endothelial cell percentage and tumor angiogenesis in a transgenic mouse model of breast cancer (Liu et al., 2010). Obesity was associated with increased IL-6 production from adipocytes and myeloid cells within tumors in murine breast cancer model (Incio et al., 2018). Inhibition of IL-6 normalized tumor vasculature, reduced hypoxia, and restored sensitivity to anti-VEGF therapy.

### Increased Pericyte Coverage of the Tumor Vasculature

Heterogeneous pericyte coverage has been described in several types of tumors, at different stages of tumor progression, and even within a single tumor stage (Hida et al., 2013). The reduction in tumor vascularity induced by anti-VEGF therapy enhances the recruitment of pericytes to maintain blood vessel function and integrity (Bergers and Hanahan, 2008). Increased pericyte coverage of these blood vessels supports tumor endothelium to survive and function despite the anti-angiogenic drug (Bergers and Hanahan, 2008; Lord and Harris, 2010). In addition, pericytes can release pro-angiogenic factors in response to PDGF (Lord and Harris, 2010). In the breast cancer vasculature, heterogenous pericyte coverage was identified (Kim et al., 2016). However, the impact of pericyte on resistance to anti-VEGF therapy in breast tumors is largely unknown.

### Angiogenesis-Independent Tumor Growth

Vasculogenic mimicry and vessel co-option may decrease the dependence on classical angiogenesis by tumors (Schneider and Miller, 2005; Bergers and Hanahan, 2008; Carmeliet and Jain, 2011). These alternative mechanisms render tumors insensitive to anti-angiogenic agents by allowing tumors to obtain the necessary blood supply when classical angiogenesis is limited (Schneider and Miller, 2005; Haibe et al., 2020). Vasculogenic mimicry is associated with aggressive breast cancer phenotypes and poor prognosis (Shen et al., 2017; Haibe et al., 2020). Bevacizumab failed to inhibit vasculogenic mimicry in the HCC1937 breast cancer cell line (Dey et al., 2015). Besides, Sun et al. showed that the administration of sunitinib induced vasculogenic mimicry in animal models of TNBC which ultimately promoted resistance to sunitinib therapy (Sun et al., 2017). Vascular co-option is another mechanism to escape angiogenesis inhibitors and has been shown to drive brain metastasis of breast cancer cells (Ramadan et al., 2020).

### Microvascular Heterogeneity

Growing evidence supports the concept of the heterogeneity of the endothelium of vessels involved in angiogenesis (Hida et al., 2013). Hida et al. showed that tumor blood vessels are heterogeneous and that tumor-associated endothelial cells had relatively large, heterogeneous nuclei, cell aneuploidy, and chromosomal alterations indicative of cytogenetic abnormalities (Hida et al., 2004; Akino et al., 2009). Altered gene and protein expression profiles in tumor endothelium have also been reported (Aird, 2012). The heterogeneity of tumor endothelial cells may differ by tumor type, tumor microenvironment, and the stage of tumor growth (Hida et al., 2013). Grange et al. showed that breast cancer-derived endothelial cells did not undergo normal cell senescence in culture, had increased motility, and constantly expressed markers of endothelial activation and angiogenesis (Grange et al., 2006). These endothelial cells were resistant to the cytotoxic activity of chemotherapeutic drugs as compared to normal micro-endothelial cells (Grange et al., 2006). The functional abnormalities of tumor-associated endothelial cells and the microvascular heterogeneity could explain, at least in part, the reduced efficacy of anti-angiogenic therapy in breast cancer by enabling endothelial cells an increased pro-angiogenic activity to acquire drug resistance (Grange et al., 2006; Madu et al., 2020).

### Tumor Heterogeneity

Lack of response to angiogenesis inhibitors may be explained in terms of the stage of progression, treatment history, and genomic constitution that exist in the tumor microenvironment (Bergers and Hanahan, 2008). An analysis of human breast cancer biopsies demonstrated a plethora of pro-angiogenic factors in late-stage breast cancers including FGF-2, in contrast to earlier-stage tumors which preferentially expressed VEGF (Relf et al., 1997). Thus, resistance to anti-VEGF drugs in advanced-stage breast cancer may be explained by the dominance of FGF-2 and other pro-angiogenic factors in such stage of the disease (Bergers and Hanahan, 2008). Invasive cancers commonly express multiple angiogenic factors and this heterogeneity occurs at an early point in time. Genetic instabilities in the tumor cells may cause alterations of both the amount and type of pro-angiogenic factors expressed in a tumor which could further promote resistance to anti-angiogenic treatments (Schneider and Miller, 2005).

### Trans-Differentiation of Cancer Stem Cells to Endothelial Cells

Cancer stem cells are a subpopulation of cancer cells capable of self-renewal, differentiation, and induction of tumorigenesis, metastasis, and drug resistance (Li et al., 2021). The potential of cancer stem cell trans-differentiating into endothelial cells has been reported in a variety of solid tumors (Li et al., 2021). Bussolati et al. showed that breast cancer stem cells were able to differentiate into the endothelial lineage in the presence of VEGF (Bussolati et al., 2009). The stem cells acquired several endothelial markers and organized into capillary-like structures forming vessels in a xenograft animal model (Bussolati et al., 2009). Similarly, Wang et al. showed that breast cancer stem cells may trans-differentiate into endothelial cells that can form capillary-like vascular structures in the cell culture system and participate in tumor angiogenesis (Wang et al., 2017). An earlier study demonstrated that microRNA-27a (miRNA-27a) expression promoted tumor angiogenesis and metastasis *in vivo* by mediating endothelial trans-differentiation of breast cancer stem-like cells (Tang et al., 2014). Brossa et al. reported the ability of breast cancer stem cells to trans-differentiate to endothelial cells expressing endothelial markers under hypoxic conditions *in vitro* (Brossa et al., 2015). Notably, treatment with the VEGFR inhibitor sunitinib but not the VEGF inhibitor bevacizumab impaired the endothelial differentiation ability of breast cancer stem cells both *in vitro* and *in vivo*. Mechanistically, sunitinib, but not bevacizumab, suppressed HIF-1α required for endothelial differentiation under hypoxic conditions (Brossa et al., 2015). Together, increasing evidence suggests that cancer stem cell endothelial trans-differentiation supports tumor vascularization and partly contributes to the failure of anti-angiogenic drugs.

## Non-VEGF Angiogenic Pathways in Breast Cancer

The lack of efficacy of the conventional angiogenesis inhibitors necessitates exploring novel angiogenic pathways in breast cancer. Given the heterogeneity of breast cancer and the complexity of angiogenesis, it is unlikely that the identification of a single target such as VEGF would be adequate in the treatment of this disease. The following section summarizes preclinical findings regarding non-VEGF/VEGFR angiogenic pathways and drugs that target them.

### Interleukins

Interleukins (ILs) are a family of cytokines known to play essential roles in the regulation of several immune cell functions such as differentiation, activation, proliferation, migration, and adhesion (Turner et al., 2014). Interactions of ILs and their receptors in endothelial cells have been shown to regulate angiogenesis through pro-angiogenic and anti-angiogenic activity (Ribatti, 2019).

IL-6 is a pleiotropic cytokine that binds to its membrane-bound receptor (IL-6R) to activate a distinct JAK/STAT signaling pathway (Taher et al., 2018). Serum IL-6 levels were elevated in breast cancer patients compared to controls (Barron et al., 2017; Raghunathachar Sahana et al., 2017), and correlated with advanced stage of the disease (Raghunathachar Sahana et al., 2017). Additionally, serum IL-6 and VEGF correlated positively in breast cancer patients (Raghunathachar Sahana et al., 2017). Higher expression of IL-6R was demonstrated in clinical specimens for patients with high-grade invasive ductal carcinoma (Bharti et al., 2018). Recent evidence showed that the IL-6/IL-6R pathway is activated in hypoxic breast cancer cells and that inhibition of IL-6R using siRNA significantly blocked angiogenesis and invasion in different models (Bharti et al., 2018). IL-6R siRNA also reduced expression of MMP-2/9 in breast cancer cells (Bharti et al., 2018). A recent study by Hegde et al. showed that a crosstalk between IL-6 and VEGFR-2 signaling pathways exists in myoepithelial and endothelial cells isolated from clinical human breast tumors (Hegde et al., 2020). IL-6 epigenetically regulated VEGFR-2 expression through induction of proteasomal degradation of DNA methyltransferase 1 leading to promoter hypomethylation and angiogenic activity (Hegde et al., 2020).

IL-8 is a pro-inflammatory cytokine that exerts its biologic activity through binding to its CXCR1 and CXCR2 receptors (Waugh and Wilson, 2008). IL-8 enhanced the proliferation of cancer cells and produced a pro-angiogenic activity (Waugh and Wilson, 2008). Serum IL-8 levels were significantly higher in breast cancer patients compared with healthy subjects and were associated with advanced disease (Benoy et al., 2004). High levels of IL-8 are secreted by stromal cells into the microenvironment of breast cancer patients compared to controls (Razmkhah et al., 2010). Evidence from preclinical studies showed that IL-8 mediated invasion and angiogenesis of breast cancer cells (Lin et al., 2004). Cancer-associated adipocytes express high levels of IL-8 in breast cancer stroma thus promoting the pro-angiogenic effects of breast adipocytes (Al-Khalaf et al., 2019). In this context, IL-8-expressing adipocytes increased vascularity of tumor xenografts as indicated by increased expression of CD34, an endothelial cell marker (Al-Khalaf et al., 2019). Neutralization of IL-8 or inhibiting its target receptors had been shown to reduce breast cancer growth and angiogenesis (Lin et al., 2004). Nannuru et al. showed that silencing of CXCR2 expression reduced tumor vascularity and inhibited spontaneous lung metastasis in an orthotopic animal model of breast cancer (Nannuru et al., 2011). Further, CXCR1 blockade with the small molecule inhibitor, repertaxin reduced metastasis in an animal model of breast cancer (Ginestier et al., 2010).

### Platelet-Derived Growth Factor

The platelet-derived growth factor (PDGF) family consists of four gene products (PDGF-A, -B, -C, and -D) that are combined into five different isoforms: PDGF-AA, -BB, -CC, -DD, and -AB (Bartoschek and Pietras, 2018). These factors bind and activate their respective RTKs, PDGFR-α, and PDGFR-β. PDGF family plays a key role in a wide range of oncologic activities essential for cancer growth including angiogenesis, fibrosis, and cellular migration (Bartoschek and Pietras, 2018).

High expression of PDGFs was correlated with an advanced presentation, increased recurrence, and poor survival in patients with invasive breast cancer (Jansson et al., 2018; Bottrell et al., 2019). PDGF is an important regulator for the motility of vascular smooth muscle cells induced by breast cancer cells (Banerjee et al., 2006). Besides, the expression of HIF-1α in invasive breast cancer was significantly associated with angiogenesis and expression of PDGF-BB (Bos et al., 2005). Earlier evidence showed that PDGFRs are expressed by breast cancer cells and endothelial cells in metastatic bone lesions in animal models (Lev et al., 2005). Imatinib remarkably inhibited PDGFR activation in breast cancer cells and tumor-associated endothelial cells and reduced microvessel density in the tumors (Lev et al., 2005). Recently, Wang et al. provided evidence from cell culture and animal studies that the downregulation of PDGF-B greatly contributed to the metformin-induced vessel normalization in breast cancer (Wang et al., 2019).

### Fibroblast Gowth Factors

Fibroblast growth factors (FGFs) belong to a large family of growth factors that includes 23 members (Hui et al., 2018). FGFs are key regulators of numerous physiological processes such as angiogenesis, wound healing, and embryonic development. These functions are mediated by the binding of FGFs with their receptors (FGFRs), which belong to the RTK family (Hui et al., 2018). Growing evidence signifies the oncogenic impact of FGFs and FGFRs to promote cancer development and progression by mediating cancer cell proliferation, survival, epithelial-to-mesenchymal transition, invasion, and angiogenesis (Wesche et al., 2011).

Dysregulations of the FGF/FGFR axis have been reported in breast cancer (Navid et al., 2020). FGF/FGFR signaling induced angiogenic activity in breast cancer cells through promoting the secretion of VEGF, enhancing HIF effects, and downregulation of thrombospondin 1 (Mattila et al., 2006; Shi et al., 2007). Chen et al. showed that dipalmitoylphosphatidic acid, a bioactive phospholipid, induced anti-angiogenic activity, and inhibited tumor growth in an experimental xenograft model of breast cancer (Chen J. et al., 2018). These effects were attributed to transcriptional inhibition of FGF-1 expression leading to the downregulation of HGF (Chen J. et al., 2018). In the same context, Cai et al. showed that neutralizing FGF-2 by a disulfide-stabilized diabody inhibited tumor growth and angiogenesis in a mouse model of breast cancer (Cai et al., 2016). The antitumor activity was associated with a significant decrease in microvessel density and the number of lymphatic vessels (Cai et al., 2016). Formononetin, an FGFR-2 inhibitor, demonstrated anti-angiogenic activity in breast cancer in both *ex vivo* and *in vivo* angiogenesis assays (Wu et al., 2015). Besides, formononetin significantly inhibited angiogenesis *in vivo* by reducing microvessel density and phosphorylated FGFR-2 levels in tumor tissue (Wu et al., 2015). Recent evidence showed that FGF-2-positive tumors are resistant to clinically available drugs targeting VEGF and PDGF (Hosaka et al., 2020). The resistance is mediated by the ability of FGF-2 to recruit pericytes onto tumor microvessels through a PDGFR-β-dependent mechanism in breast cancer and fibrosarcoma models. Dual targeting of the VEGF and PDGF produced a superior antitumor effect in FGF-2-positive breast cancer (Hosaka et al., 2020).

### Angiopoietins

Angiopoietins (Angs) represent an imperative family of vascular growth factors that produce their biological effects through binding to the RTKs, Tie-1, and Tie-2 (Akwii et al., 2019). Angiopoietin-1 (Ang-1) and angiopoietin-2 (Ang-2) are best characterized for their role in angiogenesis and vascular stability (Akwii et al., 2019). Ang-1 regulates the organization and maturation of newly formed blood vessels and promotes quiescence and structural integrity of vasculature (Brindle et al., 2006). Alternatively, Ang-2 antagonizes the effects of Ang-1 resulting in vessel destabilization (Brindle et al., 2006).

Ramanthan et al. indicated that high Ang-2 gene expression in breast cancer patients was associated with reduced survival (Ramanathan et al., 2017). In addition, a strong correlation existed between Angs and VEGF genes in breast cancer tissues (Ramanathan et al., 2017). Besides, serum levels of Ang-2 were significantly higher in breast cancer patients compared to healthy control subjects. High Ang-2 serum levels had shorter survival than that of the low Ang-2 expression group (Li et al., 2015). Evidence from preclinical models also demonstrated that Ang-2 mediated initial steps of breast cancer metastasis to the brain (Avraham et al., 2014).

He et al. showed that targeting Ang-2 with miRNA-542-3p reduced tumor growth, angiogenesis, and metastasis in animal models (He et al., 2014). Besides, Wu et al. showed that oral administration of methylseleninic acid reduced microvessel density and increased pericytes coverage by inhibiting Ang-2 in a breast cancer animal model (Wu et al., 2012). Dual inhibition of VEGF-A and Ang-2 using a bispecific antibody promoted vascular regression and normalization in a model of metastatic breast cancer (Schmittnaegel et al., 2017). Dual inhibition of Ang-1 and TGF-βR2 was also shown to suppress tumor angiogenesis in breast cancer *in vivo* (Flores-Perez et al., 2016).

### Other Non-VEGF Angiogenic Factors

Notch receptors belong to a highly conserved signaling pathway that relies on cell-cell contacts to mediate a response to environmental signals in multicellular animals (Aster et al., 2017). Four different Notch receptors are expressed in humans, each is encoded by a different gene. In addition, four functional Notch ligands exist and belong to two families: members of the Delta family of ligands; Dll-1 and Dll-4, and members of the Serrate family of ligands; Jag-1 and Jag-2 (Aster et al., 2017). In breast cancer, Notch signaling promotes cell proliferation, self-renewal, anti-apoptotic effects, and angiogenesis (Aster et al., 2017; Mollen et al., 2018). Notch expression has been associated with the progression and recurrence of breast cancer (Mollen et al., 2018). Proia et al. showed that blocking Notch-1 function with a specific antibody inhibited functional angiogenesis and breast cancer growth in animal models (Proia et al., 2015).

HGF is a member of the plasminogen-related growth factor group and is a known angiogenic factor (Nakamura and Mizuno, 2010). It is primarily expressed and produced by stromal cells, such as fibroblasts in mammary tissues (Jiang et al., 2003). The angiogenic actions of HGF are mediated by binding to its RTK, MET on endothelial cells (Organ and Tsao, 2011; Zhang et al., 2018). In the activated endothelial cells, MET is upregulated thus modulating cell dissociation, motility, proliferation, and invasion (Peruzzi and Bottaro, 2006). HGF regulates VEGF expression in tumor cells promoting angiogenic activity (Matsumura et al., 2013). Earlier studies showed that targeting HGF with retroviral ribozyme transgene or HGF antagonist reduced the growth and angiogenesis of breast tumors *in vivo* (Jiang et al., 2003; Martin et al., 2003).

Syndecans are transmembrane proteoglycans composed of a core protein and a glycosaminoglycan side chain to which growth factors are attached (Szatmari and Dobra, 2013). Syndecan-1 is the major syndecan found in epithelial malignancies (Szatmari and Dobra, 2013). Syndecan-1 ligates with several pro-angiogenic factors such as VEGF, FGFs, Wnt, and HGF, which act as signaling co-receptors (Szatmari and Dobra, 2013). Expression of syndecan-1 in breast tumors was associated with adverse prognosticators, metastasis, and reduced OS in patients (Kind et al., 2019; Qiao et al., 2019; Sayyad et al., 2019). Besides, stromal syndecan-1 expression increased vessel density and area and promoted the growth and angiogenesis of triple-negative tumors *in vivo* (Maeda et al., 2006). Schönfeld et al. showed that targeting syndecan-1 with an antibody-drug conjugate reduced the growth of TNBC in animal models when combined with chemotherapy (Schonfeld et al., 2018).

## Non-VEGF Angiogenesis Inhibitors for Treatment of Breast Cancer: Updates From Clinical Trials

Several non-VEFG/VEGFR angiogenesis inhibitors are being evaluated in breast cancer in clinical settings. An open-label, phase Ib trial evaluating antitumor activity and safety of erdafitinib; a potent and selective pan-FGFR inhibitor, in combination with fulvestrant and palbociclib in patients with metastatic breast cancer is currently recruiting patients (NCT03238196). The primary objective is to determine safety and tolerability for the combination treatment of erdafitinib with targeted treatments. Futibatinib is an orally available pan-FGFR inhibitor that is currently being evaluated in a phase II trial as monotherapy and in combination with fulvestrant in patients with locally advanced or metastatic breast cancer harboring FGFR gene amplification (NCT04024436). Infigratinib, a selective pan-FGFR inhibitor, is being assessed in a phase Ib trial in combination with tamoxifen or fulvestrant/palbociclib regimen for advanced breast cancer patients with known FGFR alterations (NCT04504331). The primary outcome of the trial is to determine dose-limiting toxicities during the first two cycles of therapy while secondary outcomes involve the identification of treatment-emergent adverse events (TEAEs) and objective tumor response. Another open-label phase Ib/II study is to evaluate the FGFR inhibitor, Debio 1347, with fulvestrant in patients with FGFR-amplified hormone receptor-positive metastatic breast cancer (NCT03344536).

Rogaratinib is another novel pan-FGFR inhibitor. Rogaratinib showed broad antitumor activity in preclinical studies (Grunewald et al., 2019). The combination of rogaratinib plus palbociclib and fulvestrant is being assessed in an open-label, multicenter, prospective, phase I dose-escalation clinical trial (NCT04483505). The primary aims of the study are to assess the recommended phase II dose and the incidence of TEAEs for the combination treatment in patients with metastatic hormone receptor-positive breast cancer who have FGFR-positive tumors. Additionally, a phase II study is assessing the long-term efficacy and tolerability of rogaratinib in patients who have received the drug in a previous clinical trial and are currently in the continuation phase (NCT04125693). The selective FGFR-2 inhibitor, RLY-4008, is being evaluated for tolerability and antineoplastic activity in several advanced solid cancers, including the breast (NCT04526106). Similarly, the pharmacological activity and tolerability of the FGFR-2 inhibitor pemigatinib are being investigated as monotherapy or in combination with other anticancer drugs in patients with advanced tumors including breast cancer in phase I/II study (NCT02393248). The primary outcomes of the study are to determine the maximum tolerated dose of pemigatinib and to assess the pharmacodynamics of the drug.

Trebananib (AMG 386) is a selective Ang-1/2-neutralizing peptibody and is the first drug to target the Angs/Tie-2 signaling pathway (Neal and Wakelee, 2010). The I-SPY 2 trial is investigating the effect of trebananib alone or in combination with standard targeted treatments in neoadjuvant settings in patients with breast cancer (NCT01042379). The KEYNOTE A60 is a multicenter, open-label phase Ib/IIa study of efineptakin alfa (NT-I7, a long-acting IL-7 agonist) in combination with pembrolizumab in patients with refractory advanced solid tumors (NCT04332653). The main outcome of the trial is to determine the safety and tolerability of NT-I7 in combination with pembrolizumab. In addition, pegilodecakin, a long-acting recombinant pegylated IL-10, has been evaluated in dose escalation/expansion study in patients with advanced solid tumors as a monotherapy or combination with other anticancer drugs in a phase I trial (NCT02009449).

Bintrafusp alfa is a first-in-class bifunctional fusion protein targeting TGF-β and programmed death-ligand 1 (PD-L1) (Paz-Ares et al., 2020). A phase I trial is recruiting breast cancer patients with stage II/III HER2-positive disease to assess the safety and tolerability of bintrafusp alfa and to evaluate the change in the percentage of tumor-infiltrating lymphocytes post-therapy (NCT03620201). Furthermore, bintrafusp alfa is being assessed as monotherapy in phase II, multicenter, open-label study in participants with TNBC (NCT04489940). Another fusion protein targeting PD-L1 and TGF-β, SHR1701, is being investigated in a phase II trial in combination with cyclin-dependent kinase 4/6 inhibitor in patients with hormone receptor-positive, HER2-negative, endocrine-resistant advanced breast cancer (NCT04355858). PF-06952229, an inhibitor of TGF-βR1, is being evaluated in a phase I dose-escalation study for its safety, tolerability, and pharmacokinetics in patients with advanced solid tumors (NCT03685591). Table 2 summarizes ongoing clinical trials for selected non-VEGF angiogenic inhibitors in breast cancer.

**TABLE 2:** Ongoing clinical trials for novel non-VEGF/VEGFR angiogenesis inhibitors in breast cancer (retrieved from: www.clinicaltrials.gov).

Clinical trial identifier	Phase	Status	Treatment	Objectives
FGFR inhibitors				
NCT03238196	Ib	Active, not recruiting	Erdafitinib, fulvestrant, and palbociclib	Safety, tolerability, and antitumor activity
NCT02052778	I/II	Recruiting	Futibatinib	Safety, tolerability, and antitumor activity
NCT04024436	II	Recruiting	Futibatinib and fulvestrant	Efficacy and safety
NCT04504331	I	Recruiting	Infigratinib and tamoxifen, or fulvestrant and palbociclib	Identify dose-limiting toxicity
NCT03344536	I/II	Completed	Debio 1347 and fulvestrant	Efficacy and dose-limiting toxicity
NCT04483505	I	Recruiting	Rogaratinib, fulvestrant, and palbociclib	Identify the recommended dose and safety
NCT04526106	I	Recruiting	RLY-4008	Maximum tolerated dose and tolerability
NCT04125693	II	Completed	Rogaratinib	Safety and tolerability
NCT02393248	I/II	Active, not recruiting	Pemigatinib and anticancer drugs	Maximum tolerated dose and efficacy
Angiopoietin inhibitors				
NCT01042379	II	Recruiting	Trebananib and standard therapies	Efficacy of treatment
Interleukin agonists				
NCT04332653	I/II	Recruiting	Efineptakin alfa and pembrolizumab	Safety and tolerability
NCT02009449	I	Active, not recruiting	Pegilodecakin and anticancer drugs	Safety and tolerability
TGF-β/TGF-βR inhibitors				
NCT03620201	I	Recruiting	Bintrafusp alfa	Impact on infiltrating lymphocytes
NCT04489940	II	Active, not recruiting	Bintrafusp alfa	Efficacy
NCT04355858	II	Recruiting	SHR1701 and cyclin-dependent kinase 4/6 inhibitor	Achieving complete or partial remission
NCT02947165	I	Recruiting	NIS793 and anti-PD-1 antibody	Safety and tolerability
NCT03685591	I	Recruiting	PF-06952229, palbociclib, and letrozole, or enzalutamide	Safety and tolerability

FGFR, fibroblast growth factor receptor; TGFβR, transforming growth factor *β* receptor; VEGF, vascular endothelial growth factor; VEGFR, vascular endothelial growth factor receptor.

## Future Perspectives and Novel Anti-Angiogenic Approaches

Targeting classical angiogenic pathways using inhibitors of VEGF/VEGFR had constantly produced suboptimal results in breast cancer. Therefore, exploring novel anti-angiogenic therapeutic approaches is of paramount importance for the treatment of aggressive and advanced breast tumors. Such approaches include vascular normalization by targeting pericytes, utilization of miRNAs and extracellular tumor-associated vesicles, using immunotherapeutic drugs, and nanotechnology.

### Targeting Pericytes

A potential strategy to sensitize tumor endothelium to angiogenesis inhibitors is by targeting pericytes to achieve tumor vascular normalization (Lord and Harris, 2010; Meng et al., 2015; Zirlik and Duyster, 2018). Normalization of tumor vasculature prevents cancer cell metastasis, improves the delivery of systemic anticancer therapies, increases the efficacy of local therapies, and enhances recognition by the host immune system. Pericyte coverage of tumor blood vessels is heterogeneous. In certain tumors, high pericyte coverage of the tumor vasculature causes resistance to anti-angiogenic therapies. Alternatively, low pericyte coverage detected in the vasculature of certain tumors reduces vascular stability and increases vascular permeability which impairs the delivery of anticancer therapies to tumor cells and allows them to metastasize (Meng et al., 2015). Earlier studies showed that combining VEGFR and PDGFR inhibitors targeting endothelial cells and pericytes, respectively, improved the efficacy of anti-angiogenic therapy and reduced tumor growth in animal tumor models (Bergers et al., 2003; Erber et al., 2004). In a xenograft model of breast carcinoma, tumor vascularization was enhanced by increasing the pericyte-endothelium association via a mechanism involving the TGF-β-fibronectin axis (Zonneville et al., 2018). In addition, Keskin et al. showed that pericyte targeting in established mouse breast tumors increased Ang-2 expression and that targeting Ang-2 signaling along with pericyte depletion restored vascular stability and decreased tumor growth and metastasis (Keskin et al., 2015). Although data from preclinical studies showed that pericyte targeting could be a novel strategy to normalize tumor vasculature, this strategy should be carefully considered as lack of pericyte coverage may disrupt vascular integrity and promote cancer metastasis (Lord and Harris, 2010; Zirlik and Duyster, 2018). Assessment of pericyte coverage of tumor vasculature and the identification of the appropriate pericyte-targeted therapy are potential challenges to pericyte targeting (Meng et al., 2015).

### MicroRNAs and Extracellular Vesicles

MicroRNAs (miRNAs) are critical regulators of signaling pathways involved in angiogenesis and cancer metastasis by interacting with the target mRNAs (Gallach et al., 2014). To date, there are groups of well-characterized miRNAs implicated in regulating endothelial cell function and angiogenesis, making them attractive targets in tumor angiogenesis (Gallach et al., 2014). Liang et al. showed that miRNA-153 suppressed breast tumor angiogenesis through targeting HIF-1α and Ang-1 in breast cancer cell lines and animal model. MiRNA-153 inhibited the proliferation, migration, and tube formation of endothelial cells and decreased the microvessel density (Liang et al., 2018a; Liang et al., 2018b). Lu et al. reported that miRNA-140-5p inhibited tumor invasion and angiogenesis by silencing VEGF-A in breast cancer cells both *in vitro* and *in vivo* (Lu et al., 2020). MiRNA-29b inhibited proliferation, migration, and tube formation of endothelial cells. Systemic administration of miRNA-29b potently suppressed breast tumor growth and vascularization by targeting Akt and downregulating VEGF and c-Myc in breast cancer cells (Li et al., 2017). Mimics of miRNA-497 suppressed the proliferation and tube formation of endothelial cells *in vitro* (Wu et al., 2016). Moreover, the overexpression of miRNA-497 reduced VEGF and HIF-1α protein levels and suppressed angiogenesis *in vivo* (Wu et al., 2016). Zou et al. showed that miRNA-145 inhibited growth and angiogenesis of TNBC *in vivo* via post-transcriptional regulation of N-Ras and VEGF (Zou et al., 2012).

Importantly, miRNAs can be transported between cancer cells and stromal cells through extracellular vesicles known to mediate cell-to-cell communication in the tumor microenvironment (Kuriyama et al., 2020). Extracellular vesicles are classified into exosomes, microvesicles, and apoptotic bodies based on the size or biogenesis of the vesicles (Kuriyama et al., 2020). Under hypoxic conditions, tumor cells release extracellular vesicles to a larger extent compared to cells in a normoxic environment (Kuriyama et al., 2020). Growing evidence points to the role of tumor-derived extracellular vesicles in tumor angiogenesis of breast cancer. Lu et al. recently reported that extracellular vesicles derived from breast cancer cells are highly enriched with miRNA-182-5p which enhanced proliferation and migration of endothelial cells *in vitro* and angiogenesis and metastasis of breast cancer *in vivo* (Lu et al., 2021). Microvesicles rich in a special VEGF isoform activated VEGFR and induced angiogenesis while being resistant to bevacizumab (Feng et al., 2017). Exosome-mediated transfer of breast cancer-secreted miRNA-105 efficiently destroyed tight junctions in endothelial monolayers associated with increased vascular permeability (Zhou et al., 2014). Few studies showed that extracellular vesicles can be targeted to prevent breast cancer metastasis and restore the activity of anti-angiogenic drugs (Zhou et al., 2014; Feng et al., 2017). Aslan *et al.* showed that docosahexaenoic acid decreased the expression of pro-angiogenic genes including HIF-1α, TGF-β, and VEGFR in breast cancer cells and their secreted exosomes (Aslan et al., 2020). Also, docosahexaenoic acid altered miRNA content in breast cancer cells and their derived exosomes in favor of the inhibition of angiogenesis (Aslan et al., 2020). Taken together, miRNAs and extracellular vesicles can be selectively targeted to reduce vascularization in breast cancer providing a novel approach for angiogenesis inhibition (Gallach et al., 2014).

### Immunotherapeutic Drugs

Normal vasculature is needed for immunosurveillance and efficient detection and killing of cancer cells by immune cells. Disorganized tumor vessels create a selective immune cell barrier limiting the extravasation of immune cells, particularly the cytotoxic T lymphocytes into blood vessels and tumor tissue (Yang et al., 2021). Further, hypoxia in the tumor microenvironment promotes lactate accumulation, extracellular acidosis, VEGF overexpression, and VEGFR activation, all of which are known drivers of immune cell tolerance and immunosuppressive status (Mendler et al., 2012; Vaupel and Multhoff, 2017). Endothelial cells are the first to come into contact with immune cells while infiltrating from the circulation into the tumor tissue (Solimando et al., 2020). Interestingly, tumor endothelial cells expressed PD-L1 and produced immunosuppressive activity contributing to tumor immune evasion in a mouse model of melanoma (Taguchi et al., 2020). Further, leukocyte adhesion was remarkably diminished in tumor vessels (Dirkx et al., 2003). Tumors secrete angiogenic growth factors that can downregulate endothelial adhesion molecules essential for the interactions with granulocytes, macrophages, and natural killer cells on the vascular endothelium (Griffioen, 2008). The suppression of these selective adhesion molecules leads to the loss of the adhesive properties of the tumor endothelium thereby impairing immune cell infiltration to tumor tissues. Solimando et al. showed that junctional adhesion molecule-A (JAM-A) is an important factor influencing angiogenesis and extra-medullary dissemination in patients with multiple myeloma and its targeting suppressed multiple myeloma-associated angiogenesis both *in vitro* and *in vivo* (Solimando et al., 2019; Solimando et al., 2021). Bednarek et al. recently demonstrated that targeting JAM-A with an antagonistic peptide inhibited the adhesion and trans-endothelial migration of breast cancer cells (Bednarek et al., 2020). In breast cancer, vascular cell adhesion molecule-1 was aberrantly expressed and mediated angiogenesis and metastasis by binding to its ligand α4β1integrin (Sharma et al., 2017). Earlier findings also showed that angiogenic stimuli in the microenvironment of breast cancer may influence the expression of endothelial adhesion molecules to prevent leukocyte infiltration to tumor tissue (Bouma-Ter Steege et al., 2004). Dual VEGF/Ang-2 inhibition normalized tumor vasculature and reprogrammed the tumor immune microenvironment toward the antitumor phenotype in an animal model (Kloepper et al., 2016). Therefore, selective targeting of adhesion molecules and normalizing tumor vasculature could improve immune cell endothelial adhesion and strengthen the antitumor immune response in epithelial tumors, including breast cancer.

A growing body of evidence describes the interplay between immune cells and vasculature in the tumor microenvironment. The immune response and vascular normalization seem to be mutually regulated (Fukumura et al., 2018; Huang et al., 2018). Normalization of the tumor vasculature improves the infiltration of immune effector cells into tumors enhancing antitumor immune activity (Fukumura et al., 2018; Solimando et al., 2020; Yang et al., 2021). Likewise, immunotherapy can promote vascular normalization which further improves the effectiveness of immunotherapeutic drugs and response to anti-angiogenic therapies (Huang et al., 2018; Ciciola et al., 2020; Yang et al., 2021). In preclinical models of breast cancer, immune checkpoint inhibitors induced normalization of tumor vasculature and increased infiltration of immune cells into breast tumors (Tian et al., 2017; Zheng et al., 2020). Together, the combination of anti-angiogenic and immunotherapeutic drugs might be an attractive approach to increase the effectiveness of each class of drugs and reduce the emergence of drug resistance (Fukumura et al., 2018; Huang et al., 2018; Solimando et al., 2020). The combination treatment has shown encouraging results in various cancer types (Ciciola et al., 2020; Madu et al., 2020). In a preclinical study, Allen et al. revealed that treatment with a combination of anti-VEGFR-2 and anti-PD-L1 antibodies sensitized tumors to anti-angiogenic therapy and prolonged its efficacy in breast cancer (Allen et al., 2017). Li et al. recently demonstrated a dose-dependent synergism for the combined treatment of anti-angiogenic therapy and immune checkpoint blockade (Li et al., 2020). In this regard, the combination of low-dose anti-VEGFR2 antibody with anti-programmed cell death protein-1 (PD-1) therapy normalized tumor vasculature, induced immune cell infiltration, and upregulated PD-1 expression on immune cells in syngeneic breast cancer mouse models. Additionally, the combined treatment was effective and tolerable in patients with advanced TNBC (Li et al., 2020). An open-label, randomized, parallel, phase II trial investigated the combination treatment of apatinib, a VEGFR-2 tyrosine kinase inhibitor with the anti-PD-1 monoclonal antibody camrelizumab in patients with advanced TNBC (Liu et al., 2020). The results showed that the combination treatment produced favorable therapeutic outcomes in terms of improved objective response rate and PFS which was associated with increased tumor-infiltrating lymphocytes. The adverse events were manageable and included elevated aminotransferases and hand-foot syndrome (Liu et al., 2020). Multiple clinical trials of combining anti-angiogenic therapy and immune checkpoint inhibitors are underway (Zirlik and Duyster, 2018).

### Nanotechnology

The nanotechnology-based approach is an emerging strategy for the development of therapies targeting tumor angiogenesis which could improve the current pharmacokinetic profiles of anti-angiogenic drugs and favor their selective accumulation in tumors (Banerjee et al., 2011; Darweesh et al., 2019). Compared to the free drug, *in vitro* and *in vivo* assays showed that gold nanoparticle-conjugated quercetin inhibited angiogenesis and invasion of breast cancer by targeting the EGFR/VEGFR-2 signaling pathway (Balakrishnan et al., 2016). Radical-containing nanoparticles produced *in vitro* and *in vivo* anti-angiogenic activity in a breast cancer model that was mediated by suppressing VEGF in cancer cells (Shashni et al., 2021). Nanoparticles were also utilized to deliver a combination of therapy for breast cancer to produce anticancer and anti-angiogenic activity (Zhao et al., 2017). In a recent study by Gong et al., nanoparticles delivering an inhibitor of sphingosine-1 phosphate receptor-1 dramatically inhibited TNBC growth and angiogenesis *in vivo* via downregulating STAT3/VEGF axis (Gong et al., 2021). Table 3 provides a list of novel approaches for targeting vascular growth and angiogenesis in breast cancer.

**TABLE 3:** Novel targets and/or strategies for the inhibition of angiogenesis in breast cancer.

Target/strategy	Mode of action	Outcome	Evidence	Refence
Non-angiogenic vascular growth				
Vasculogenesis	Inhibiting TAMs recruiting cytokines with neutralizing antibodies	Reduced tumor vascularization and improved sensitivity to bevacizumab	Preclinical	Longatto Filho et al. (2010)
	Inhibiting SDF1 with a neutralizing antibody	Decreased infiltration of myeloid cells, reduced endothelial cell percentage, and tumor angiogenesis	Preclinical	Liu et al. (2010)
Cancer stem cell trans-differentiation into endothelial cells	Inhibition of VEGFR with sunitinib	Blocked endothelial differentiation of cancer stem cells by suppressing HIF-1α	Preclinical	Brossa et al. (2015)
Non-VEGF/VEGFR angiogenic factors				
ILs	Inhibition of IL-6 with a neutralizing antibody	Normalized tumor vasculature and restored sensitivity to anti-VEGF therapy	Preclinical	Incio et al. (2018)
	Inhibition of IL-6R with siRNA	Blocked angiogenesis by reduced expression of MMP and HIF-1α	Preclinical	Bharti et al. (2018)
	Neutralization of IL-8 or inhibiting its receptors	Reduce tumor growth and angiogenesis	Preclinical	Lin et al. (2004)
	Inhibition of IL-8R (CXCR2)	Reduced tumor vascularity and inhibited spontaneous lung metastasis	Preclinical	Nannuru et al. (2011)
	Inhibition of IL-8R (CXCR1) with the small molecule repertaxin	Reduced metastasis	Preclinical	Ginestier et al. (2010)
PDGFR	Inhibition of PDGFR with imatinib	Reduced microvessel density in tumors	Preclinical	Lev et al. (2005)
FGF	Dipalmitoylphosphatidic acid-induced inhibition of FGF-1 expression and downregulation of HGF	Inhibition of tumor growth and angiogenesis	Preclinical	Chen et al. (2018a)
	Neutralizing FGF-2 by a disulfide-stabilized diabody	Inhibition of tumor growth, angiogenesis, and decreased microvessel density	Preclinical	Cai et al. (2016)
FGFR	Inhibition of FGFR-2 by formononetin	Reducing microvessel density and inhibition of angiogenesis	Preclinical	Wu et al. (2015)
Angs	Targeting Ang-2 with miRNA-542-3p	Reduced tumor growth, angiogenesis, and metastasis	Preclinical	He et al. (2014)
	Inhibition of Ang-2 by methylseleninic acid	Reduced microvessel density and increased pericytes coverage	Preclinical	Wu et al. (2012)
Notch	Inhibition of Notch-1 function with a specific antibody	Inhibition of tumor growth and angiogenesis	Preclinical	Proia et al. (2015)
HGF	Inhibition of HGF with retroviral ribozyme transgene	Reduced tumor growth and angiogenesis	Preclinical	Jiang et al. (2003)
	Inhibition of HGF with the antagonist, NK4	Reduced tumor growth and angiogenesis	Preclinical	Martin et al. (2003)
Novel approaches				
Pericytes	Inhibition of Ang-2 signaling with pericyte depletion	Restored vascular stability and decreased tumor growth and metastasis	Preclinical	Keskin et al. (2015)
MicroRNAs	MiRNA-153	Reduced endothelial cell migration and tube formation, microvessel density, and angiogenesis through targeting HIF-1α and Ang-1	Preclinical	Liang et al. (2018a)
				Liang et al. (2018b)
	MiRNA-140-5p	Inhibited tumor invasion and angiogenesis by silencing VEGF	Preclinical	Lu et al. (2020)
	MiRNA-29b	Inhibited tube formation of endothelial cells and tumor vascularization by downregulating VEGF and c-Myc	Preclinical	Li et al. (2017)
	MiRNA-497 mimics	Suppressed tube formation of endothelial cells and inhibition of angiogenesis by targeting VEGF and HIF-1α	Preclinical	Wu et al. (2016)
	MiRNA-145	Inhibited tumor growth and angiogenesis via post-transcriptional regulation of N-Ras and VEGF	Preclinical	Zou et al. (2012)
Extracellular vesicles	Docosahexaenoic acid decreased pro-angiogenic factors and altering miRNAs in cancer cell-secreted exosomes	Inhibition of angiogenesis	Preclinical	Aslan et al. (2020)
Adhesion molecules	Inhibition of JAM-A with an antagonistic peptide	Reduced cancer cell adhesion and *trans*-endothelial migration	Preclinical	Bednarek et al. (2020)
Immunotherapy	Combination of anti-VEGFR-2 and anti-PD-L1 antibodies	Sensitized tumors to anti-angiogenic therapy	Preclinical	Allen et al. (2017)
	Combination of anti-VEGFR2 and anti-PD-1 antibodies	Normalization of tumor vasculature and induced immune cell infiltration	Preclinical Clinical	Li et al. (2020)
	Combination of VEGFR-2 tyrosine kinase inhibitor and anti-PD-1 antibody	Increased tumor-infiltrating lymphocytes	Clinical	Liu et al. (2020)
Nanotechnology	Gold nanoparticle-conjugated quercetin	Inhibited angiogenesis and invasion by targeting EGFR/VEGFR-2 pathway	Preclinical	Balakrishnan et al. (2016)
	Radical-containing nanoparticles	Anti-angiogenic activity mediated by suppressing VEGF in cancer cells	Preclinical	Shashni et al. (2021)
	Nanoparticles delivering sphingosine-1 phosphate receptor-1 inhibitor	Inhibition of tumor growth and angiogenesis via downregulating STAT3/VEGF axis	Preclinical	Gong et al. (2021)

Angs, angiopoietins; FGF, fibroblast growth factor; FGFR, fibroblast growth factor receptor; HGF, hepatocyte growth factor; HIF-1α, hypoxia-inducible factor-1α; JAM-A, junctional adhesion molecule-A; ILs, interleukins; ILR, interleukin receptor; MiRNA, microRNAs; MMP, matrix metalloproteinases; PD-1, programmed cell death protein-1; PDGFR, platelet-derived growth factor receptor; PD-L1, programmed death-ligand 1; SDF1, stromal cell-derived factor-1; TAMs, tumor-associated macrophages; VEGF, vascular endothelial growth factor; VEGFR, vascular endothelial growth factor receptor.

## Conclusion

Breast cancer is a notable example where anti-angiogenic agents had constantly failed to make a significant impact on the survival of patients in clinical settings. One essential aspect to improve the efficacy of clinically available anti-angiogenic drugs is to better understand the vascular biology of breast cancer at the different stages and molecular types of the disease. Besides, a greater understanding of the adaptive and intrinsic resistance mechanisms would enhance the proper utilization of angiogenesis inhibitors. Further evaluation for the role of stromal cells within the tumor microenvironment in mediating resistance to anti-angiogenic drugs will improve the efficacy and durability of anti-angiogenic therapy. Another important facet to consider for the limited activity of angiogenesis inhibitors in breast cancer is the population under examination to allow the identification of breast cancer patients who would benefit most from anti-angiogenic drugs. Alongside, research should continue to explore the role of non-VEGF/VEGFR signaling pathways in the vascularization of breast cancer to develop clinically useful therapeutic targets. Furthermore, there are several ongoing efforts to describe novel strategies to inhibit tumor angiogenesis through pericyte targeting, the use of immunotherapy, miRNAs, and the implementation of nanotechnology. Despite the preclinical success of many of these strategies, limited clinical evidence is available to support their implementation in breast cancer treatment.
